# Severe neonatal dengue with confirmed vertical transmission of DENV-3: A fatal case from Honduras

**DOI:** 10.1016/j.idcr.2025.e02423

**Published:** 2025-11-08

**Authors:** Camilo Ernesto Palma Hidalgo, Luis Enrique Romero Reyes, Sara Elizabeth Milla Salguero, Eduardo Smelin Perdomo Domínguez

**Affiliations:** a Hospital Dr. Mario Catarino Rivas, San Pedro Sula, Honduras; b Department of Pediatrics and Infectious Diseases, Hospital Dr. Mario Catarino Rivas, San Pedro Sula, Honduras; c Clínica Médica Villanueva, Villanueva, Honduras

**Keywords:** Dengue, Neonate, Vertical transmission, Pregnancy

## Abstract

Dengue virus (DENV) infection is a major public health concern, with an estimated 390 million infections annually. Although typically self-limiting, dengue can progress to severe, life-threatening complications in high-risk groups such as neonates. Vertical transmission has been reported in up to 22.7 % of pregnancies complicated by maternal dengue. We report a fatal case of severe neonatal dengue due to DENV-3, confirmed by RT-PCR in both mother and infant. The neonate, born at 38 weeks of gestation via cesarean section to a mother with severe dengue and preeclampsia with severe features, complicated by HELLP syndrome, developed hemodynamic instability and died on day 11 of life. The mother also died shortly after delivery despite intensive management. This case highlights the diagnostic and therapeutic challenges of congenital dengue, especially in resource-limited settings. The presence of DENV-3 in both mother and neonate, with fatal outcomes in both, highlights the need for early recognition, timely intensive care, and further investigation into serotype-specific risks.

## Introduction

Dengue is a major public health concern in tropical and subtropical regions, with an estimated 390 million infections annually [1]. Although typically self-limiting, dengue often presents with non-specific symptoms, making early diagnosis difficult. While most infections are mild or asymptomatic, some progress to severe forms particularly in high-risk populations [2].

Neonates are particularly vulnerable when vertical transmission occurs near delivery, as clinical features often resemble neonatal sepsis, complicating timely diagnosis. Vertical transmission has been estimated to occur in 18.5–22.7 % of pregnancies affected by maternal dengue, and while symptomatic maternal dengue nearly doubles the risk of fetal death, severe dengue has been associated with a fivefold increase in this risk [3], [4].

Here, we report a case of severe neonatal dengue infection due to DENV-3, with vertical transmission confirmed by RT-PCR in both the mother and newborn, which resulted in shock and hemorrhage, culminating in death on day 11 of life. This case highlights the clinical and diagnostic challenges of neonatal dengue and emphasizes the importance of early recognition, especially in endemic regions with limited access to specialized care.

## Case presentation

We report the case of a Honduran female neonate born via cesarean section to non-consanguineous parents. The 23-year-old mother, previously healthy and with no underlying medical conditions, was diagnosed with severe dengue and preeclampsia with severe features, complicated by HELLP syndrome, late in pregnancy. Dengue infection was confirmed by NS1 antigen and RT-PCR, which identified DENV-3. Serological testing showed positive DENV IgG and negative IgM, consistent with a secondary infection.

The mother developed fever, headache, myalgia, arthralgia, and four episodes of epistaxis at 38 weeks of gestation, approximately four days before delivery. She was admitted on September 22, 2024, and underwent cesarean section for maternal and fetal benefit, given ongoing fever, thrombocytopenia, and elevated transaminases. Despite initial stability, her condition deteriorated after delivery, requiring ICU admission, and she died on September 26, 2024, during the postpartum period.

Due to maternal dengue infection, the neonate was admitted for close observation and supportive care. She was born at 38 weeks of gestation according to her last menstrual period and ultrasound, consistent with a term pregnancy. Postnatal evaluation using the Capurro method corresponded to 36 weeks and 4 days of gestational age, with Apgar scores of 8 and 9 at 1 and 5 min, respectively. Vital signs at birth were within normal limits, and she was alert, with normal tone and reactive to stimuli. Anthropometric measurements were as follows: weight 2120 g (low birth weight), length 42 cm, and head circumference 33 cm. Physical examination was unremarkable. Initial laboratory studies on day 1 of life showed normal hematologic parameters, and hematologic and coagulation trends during hospitalization are summarized in Table 1.

**Table 1:** Serial hematological and coagulation parameters during hospitalization.

Day of Life	Hemoglobin (g/dL)	Hematocrit (%)	Platelets (×10⁹/L)	WBC (×10⁹/L)	PT (sec)	aPTT (sec)	INR
1	15	47.5	184	8.61	—	—	—
3	17.7	53	138	7.71	14.9	46.6	1.26
5	17.3	47.7	26	30	25.2	119.3	2.14
6	15.4	42.7	20	27.4	—	—	—
9	14.5	40.4	23	18.3	13.9	42.6	1.18

Abbreviations: WBC, white blood cell count; PT, prothrombin time; aPTT, activated partial thromboplastin time; INR, international normalized ratio.

On follow-up, the infant remained active and reactive to stimuli, with an effective sucking reflex and a vigorous cry. Mucous membranes were well hydrated, fontanels were normotensive, and the rest of the physical exam was unremarkable.

On day 3 of life, she developed hemodynamic instability, presenting with lethargy, cold extremities, and delayed capillary refill. Given the maternal history of severe dengue and the evolving clinical picture, NS1 antigen testing was performed and returned positive, while IgM and IgG were negative, confirming dengue infection. Based on these findings, a diagnosis of severe neonatal dengue was established. Fluid resuscitation, empiric antibiotics, and inotropic support with dobutamine were initiated.

**Fig. 1:**
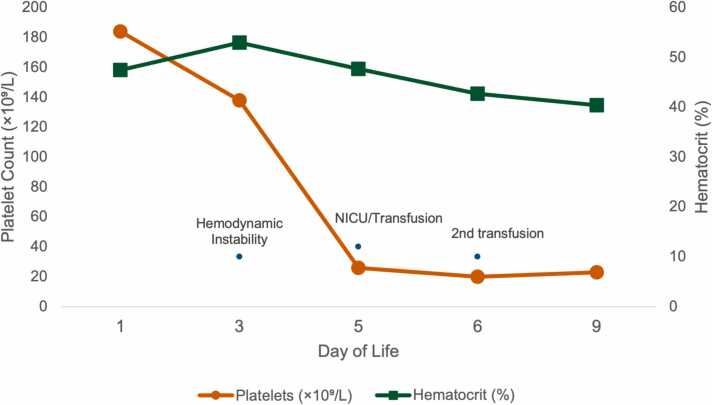
Evolution of platelet count and hematocrit during hospitalization.

Laboratory evaluation revealed progressive thrombocytopenia, hemoconcentration, and coagulopathy, as summarized in Table 1 and illustrated in Fig. 1. Despite transient hemodynamic improvement, the infant later developed worsening bleeding and gastrointestinal hemorrhage, requiring transfer to the neonatal intensive care unit (NICU) for advanced management.

In the NICU, she experienced further platelet decline and disseminated intravascular coagulation. Supportive therapy included platelet, plasma, and cryoprecipitate transfusions, as well as intravenous tranexamic acid and escalation of inotropic support. Despite maximal care, she progressed to refractory shock and multi-organ failure, and death was declared on the 11th day of life. RT-PCR confirmed DENV-3 in both maternal and neonatal samples, consistent with vertical transmission.

## Discussion

Vertical transmission of dengue virus is a recognized but still poorly elucidated phenomenon. Although its overall incidence remains uncertain, available data suggest that transmission from mother to fetus can occur both in early and late pregnancy, particularly when maternal infection coincides with the peripartum period. When maternal infection occurs within two weeks before or up to two days after delivery, the risk of vertical transmission increases to approximately 56.2 % [5]. This elevated risk is attributed to insufficient transplacental transfer of protective maternal antibodies when infection occurs too close to delivery [6]. In our case, the neonate was born only hours after maternal symptom onset, which placed her within this high-risk transmission window. Clinical deterioration occurred on day three of life, slightly earlier than the 5–13-day range reported in similar cases [6].

The present case occurred in September 2024, coinciding with an official epidemiological alert (May–September 2024) declared by the Honduran Ministry of Health following a regional warning from PAHO/WHO regarding increased dengue transmission in Central America [7]. During this period, co-circulation of all four dengue virus serotypes (DENV-1–4) was reported in Honduras, with the highest incidence in the San Pedro Sula and Cortés regions [8]. According to official statements from the Honduran Health Surveillance Unit, DENV-3 was the predominant serotype among hospitalized patients during this period. The alert remained active for four months and was subsequently lifted in October 2024 as case numbers declined.

Adverse perinatal outcomes are frequently observed in pregnancies affected by maternal dengue. Symptomatic dengue nearly doubles the odds of fetal death, while severe dengue increases this risk up to fivefold [4]. Our case aligns with this association, as both the mother and neonate succumbed to the infection. The neonate was born at term with low birth weight. Although some studies, including meta-analyses, have suggested a link between maternal dengue and preterm birth, others have found no significant association with either preterm birth or low birth weight [9], [10].

The clinical spectrum of congenital dengue is heterogeneous, ranging from mild disease to severe, life-threatening complications. Some neonates recover without significant medical support, as reported in term infants from Colombia and India who remained stable despite confirmed maternal infection or developed only transient thrombocytopenia and rash without hemodynamic compromise [11], [12]. Other reports from Chile, Vietnam, Thailand, and Sri Lanka describe severe neonatal dengue cases that responded adequately to intensive care measures. Infants with plasma leakage or coagulopathy have recovered following inotropic support, transfusions, and fluid-guided management [13], [14], [15], [16].

In contrast, some reports document fatal outcomes or prolonged complications. A preterm infant with pulmonary hemorrhage and refractory shock died despite aggressive management, while others required prolonged ventilation and transfusions but ultimately survived without sequelae [17]. A case series of seven vertically transmitted DENV-2 infections reported that, although several neonates developed severe disease requiring intensive care, all survived [18]. Compared to these findings, our case—characterized by DENV-3 infection, early critical illness, and dual maternal-neonatal mortality—emphasizes both the potential severity of congenital dengue and the consequences of delayed care escalation.

The fatal outcome in both mother and neonate was likely multifactorial, resulting from an interplay between viral and host factors. The maternal infection corresponded to a secondary DENV infection, a condition known to trigger antibody-dependent enhancement, facilitating viral entry into monocytes and macrophages, suppressing innate antiviral response, and amplifying the inflammatory cascade [19]. The transmitted serotype, DENV-3, has been linked to increased clinical virulence and severe disease in regional studies. Recent cohort data from Nicaragua further demonstrated that DENV-3 infections were significantly more likely to result in symptomatic and severe disease than other serotypes [20].

On the host side, the neonate’s immature immune system and limited transplacental antibody transfer likely predisposed to plasma leakage and coagulopathy. In addition, DENV infection during pregnancy can induce pro-inflammatory cytokines (IL-6, IL-8, TNF-α) and endothelial injury, facilitating placental infection and viral translocation. Histopathological findings such as hypoxia, choriodeciduitis, deciduitis, and intervillositis support this mechanism of vertical transmission [10].

Our experience also illustrates the systemic barriers that may influence prognosis in low-resource settings. Although transfer to NICU was eventually achieved, it was delayed due to lack of bed availability. This likely contributed to the neonate’s rapid clinical deterioration despite early diagnostic suspicion and initiation of supportive therapy. In addition, molecular genotyping of the DENV-3 isolate was not performed due to limited laboratory capacity, representing another constraint commonly encountered in low- and middle-income settings.

Future studies should aim to define serotype-specific risks, identify predictors of severe outcomes, and develop standardized protocols for diagnosing and managing congenital dengue. Such efforts are essential to raise awareness, enhance surveillance, and improve care in endemic regions.

## Ethical approval

The study was reviewed and approved by the Ethics Committee on Research of Universidad Tecnológica Centroamericana (UNITEC), IRB No. 00012967, under approval act No. 014–2024.

## Funding

This research did not receive any specific grant from funding agencies in the public, commercial, or not-for-profit sectors.

## CRediT authorship contribution statement

**Perdomo Dominguez Eduardo Smelin:** Writing – review & editing, Writing – original draft, Supervision, Conceptualization. **Sara Elizabeth Milla Salguero:** Writing – review & editing, Writing – original draft, Data curation, Conceptualization. **Luis Enrique Romero Reyes:** Data curation, Conceptualization. **Camilo Ernesto Palma Hidalgo:** Writing – review & editing, Writing – original draft, Data curation, Conceptualization.

## Declaration of Competing Interest

The authors declare that they have no known competing financial interests or personal relationships that could have appeared to influence the work reported in this paper.

## Data Availability

None.
